# Nucleophilic fluoroalkylation/cyclization route to fluorinated phthalides

**DOI:** 10.3762/bjoc.14.12

**Published:** 2018-01-19

**Authors:** Masanori Inaba, Tatsuya Sakai, Shun Shinada, Tsuyuka Sugiishi, Yuta Nishina, Norio Shibata, Hideki Amii

**Affiliations:** 1 Division of Molecular Science, Graduate School of Science and Technology, Gunma University, 1-5-1 Tenjin-cho, Kiryu, Gunma, 376-8515, Japan; 2 Research Core for Interdisciplinary Sciences, Okayama University, 3-1-1 Tsushimanaka, Kita-ku, Okayama 700-8530, Japan; 3 Department of Nanopharmaceutical Sciences, Department of Life Science and Applied Chemistry, Nagoya Institute of Technology Gokiso, Showa-ku, Nagoya 466-8555, Japan

**Keywords:** cyclization, fluorine, lactone, phthalide, trifluoromethylation

## Abstract

Discribed in this article is a versatile and practical method for the synthesis of C3-perfluoroalkyl-substituted phthalides in a one-pot manner. Upon treatment of KF or triethylamine, 2-cyanobenzaldehyde reacted with (perfluoroalkyl)trimethylsilanes via nucleophilic addition and subsequent intramolecular cyclization to give perfluoroalkylphthalides in good yields.

## Introduction

**Scheme 1:**
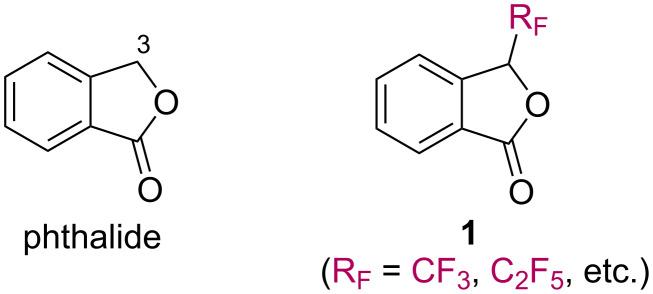
Phthalide and fluorinated phthalides (**1**).

Phthalides (1(3*H*)-isobenzofuranones) are frequently found in natural products and exhibit a range of bioactivity (Scheme 1) [1–2]. Substituted phthalides have been used as building blocks for the synthesis of useful bioactive compounds. There is a growing interest in the usefulness of phthalides and their derivatives. Organofluorine compounds often show attractive physical, chemical, and biological properties and are widely used in many fields, such as pharmaceuticals, agrochemicals, and materials [3–10]. Selective incorporation of fluorine or a fluoroalkyl group into a molecule is a topic of significant interest in organic chemistry. Fluorinated phthalides are considered to be one of the most fascinating organofluorine compounds. However, to our best knowledge, there have been few reports on the preparation of fluoroalkyl phthalides [11–19]. The first synthesis of 3-(trifluoromethyl)phthalides was accomplished by Reinecke and Chen in 1993. They studied *ortho*-lithiation of phenyloxazolines and the subsequent reactions with pentafluoroacetone and hexafluoroacetone to give 3-(trifluoromethyl)phthalide derivatives [11]. In 2006, Pedrosa et al. reported the nucleophilic trifluoromethylation of protected *ortho*-phthalaldehyde, followed by deprotection and oxidation to afford 3-(trifluoromethyl)phthalide [12]. Pohmakotr et al. demonstrated the nucleophilic trifluoromethylation of acid anhydrides to produce γ-hydroxy-γ-trifluoromethyl-γ-butyrolactones, which acted as good precursors in the synthesis of γ-trifluoromethyl-γ-butyrolactones with organometallic reagents [16]. All these protocols involve multiple steps to obtain trifluoromethylphthalides. An operational simple procedure for a short-step synthesis of 3-(trifluoromethyl)phthalide is required due to their great potential in a variety of applications. Herein, we wish to report a general and convenient synthesis of 3-(perfluoroalkyl)phthalides **1** by nucleophilic perfluoroalkylation of 2-cyanobenzaldehyde (**2**) and subsequent intramolecular cyclization.

## Results and Discussion

The reaction procedure is very simple. A mixture of 2-cyanobenzaldehyde (**2**), CF_3_–SiMe_3_ (so-called Ruppert–Prakash reagent) [20–21], and a catalytic amount of KF in anhydrous DMF was stirred at room temperature for 1 h. After work-up under acidic conditions, 3-(trifluoromethyl)-1(3*H*)-isobenzofuranone (**1a**) was obtained in 99% NMR yield (95% isolated yield) (Table 1, entry 1). As a good alternative activator of CF_3_–SiMe_3_, the use of a Lewis base such as triethylamine [22–23] worked well for the synthesis of 3-(trifluoromethyl)phthalide (**1a**, Table 1, entries 2–4). When aldehyde **2** was treated with CF_3_–SiMe_3_ in the presence of Et_3_N at 50 °C, the cascade trifluoromethylation/cyclization proceeded smoothly to afford phthalide **1a** in 70% isolated yield (Table 1, entry 4).

**Table 1:** Trifluoromethylation/cyclization of 2-cyanobenzaldehyde.

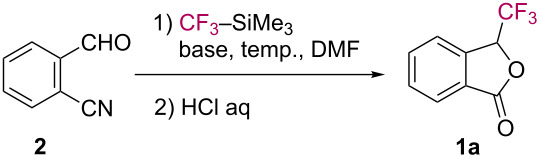			

Entry	Base (equiv)	Conditions	Yield of **1a** (%)^a,b^

1	KF (0.1)	rt, 1 h	99 (95)
2	Et_3_N (0.5)	rt, 1 h	80
3	Et_3_N (0.5)	50 °C, 1 h	86
4	Et_3_N (1.0)	50 °C, 1 h	91 (70)

^a^Yields were determined by ^19^F NMR analysis using 1,3-bis(trifluoromethyl)benzene as an internal standard. ^b^The values in parentheses indicate the isolated yield of **1a**.

**Scheme 2:**
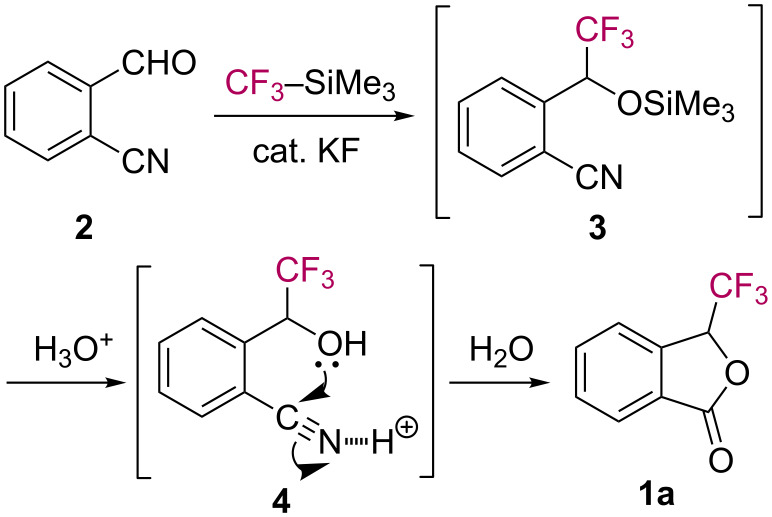
Plausible reaction mechanism for the formation of phthalide **1a**.

The formation of phthalide **1a** can be explained by assuming the pathway shown in Scheme 2. The formyl group in aldehyde **2** undergoes nucleophilic trifluoromethylation triggered by a catalytic amount of KF to give the *ortho*-cyanobenzyl silyl ether **3**. Upon treatment with aq HCl, the subsequent lactonization of **4** takes place to afford trifluoromethylphthalide **1a**.

**Scheme 3:**
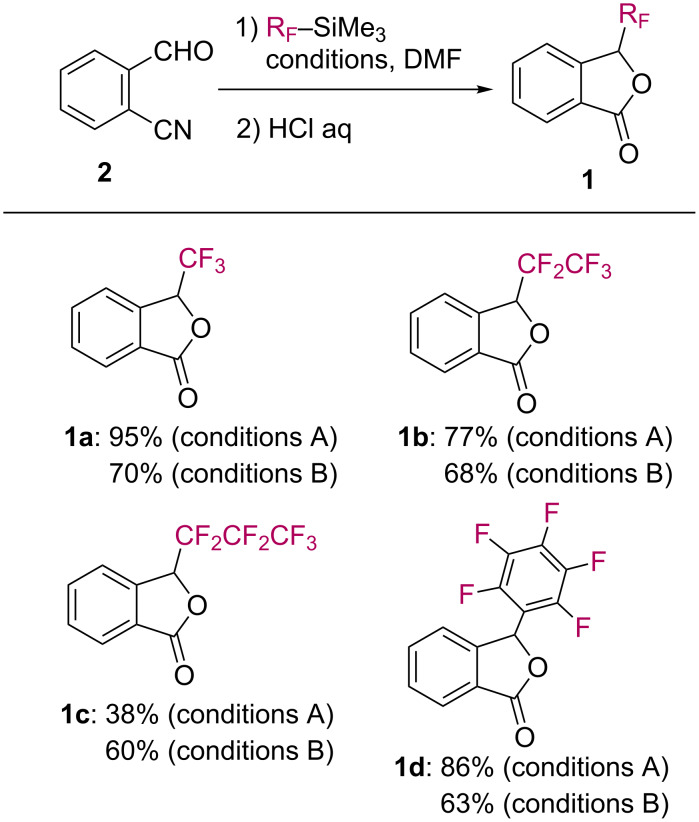
Synthesis of fluorinated phthalides **1**.

Other examples of the one-pot synthesis of fluorinated phthalides **1** are given in Scheme 3. The reactions of 2-cyanobenzaldehyde (**2**) with organosilicon compounds (R_F_–SiMe_3_) proceeded cleanly by the use of KF (conditions A) or Et_3_N (conditions B). As a consequence, pentafluoroethyl, heptafluoropropyl, and pentafluorophenyl [24–25] groups were successfully installed at the C3-position of phthalides **1b–d**.

**Scheme 4:**
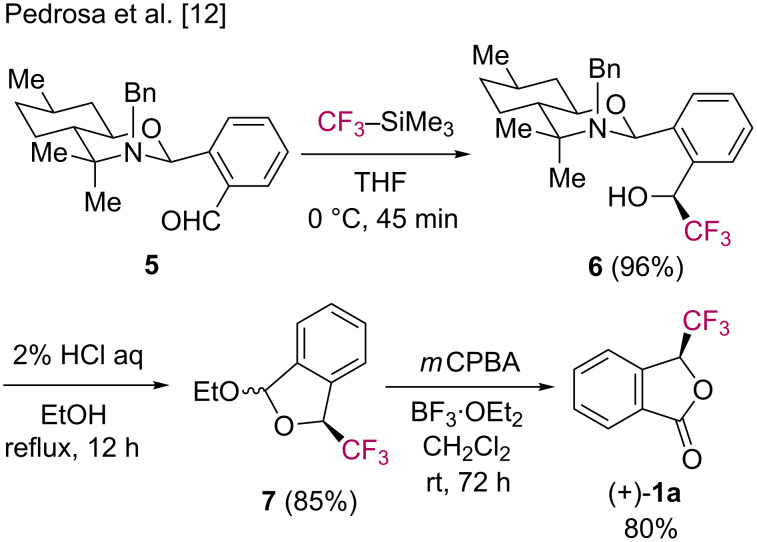
Asymmetric synthesis of **1a** using a chiral auxiliary.

Thus, the selective formation of fluorinated phthalides **1** represents a synthetic usefulness for further applications. In particular, the asymmetric synthesis of C3-substituted phthalides is of considerable importance in chemistry [26–32]. Enantioselective fluoroalkylation/lactonization reactions are worth investigating since a new stereogenic carbon center next to the fluoroalkyl groups is generated in products **1**. To the best of our knowledge, only one successful example of an asymmetric synthesis of 3-(trifluoromethyl)phthalide (**1a**) using a chiral auxiliary was published to date. In 2006, Pedrosa and co-workers discribed the diastereoselective nucleophilic trifluoromethylation of aldehyde **5**, which was prepared by condensation of *ortho*-phthalaldehyde with (−)-8-benzylaminomenthol (Scheme 4) [12]. Only diastereoisomer **6** was detected in the NMR analysis. Acid-promoted deprotection of hemiaminal **6** and subsequent oxidation of acetal **7** gave the enantiopure phthalide **1a** in good yield.

Although a high control of diastereoselectivity (using stoichiometric auxiliary strategy) was achieved for the asymmetric synthesis of trifluoromethylphthalide **1a**, it is desirable to reduce the amount of the chiral sources. Next, we undertook the development of a catalytic asymmetric synthesis of **1** in a one-pot manner. The results of our trial are summarized in Table 2.

**Table 2:** Trifluoromethylation/cyclization of 2-cyanobenzaldehyde (**2**) in the presence of chiral catalysts.

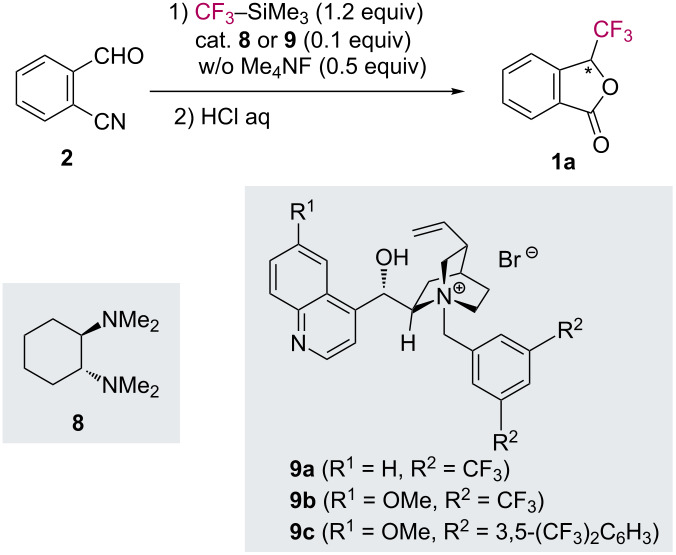					

Entry	Catalyst	Solvent	Conditions	Yield of **1a** (%)^a^	% ee^b^

1	**8**	DMF	rt, 1 h	79	0
2	**8**	DMF	0 °C, 1 h	62	0
3	**9a**	toluene/CH_2_Cl_2_ (2:1)	−60 °C, 24 h	61	0
4	**9b**	toluene/CH_2_Cl_2_ (2:1)	−60 °C, 24 h	51	12
5	**9c**	toluene/CH_2_Cl_2_ (2:1)	−60 °C, 24 h	76	6

^a^Isolated yield of **1a**. ^b^Each enantiomeric excess (ee) was determined by HPLC analyses.

For the catalytic asymmetric synthesis of **1**, we carried out the nucleophilic trifluoromethylation of **2** employing a small amount of chiral tertiary amines. However, the use of (1*R*,2*R*)-(*N*,*N*,*N’*,*N’*-tetramethyl)-1,2-diaminocyclohexane (**8**) as a chiral catalyst resulted in the formation of a racemic mixture of **1a** (Table 2, entries 1 and 2).

Previously, Shibata et al. reported a cinchona alkaloid/Me_4_NF-catalyzed nucleophilic enantioselective trifluoromethylation of carbonyl compounds [33–35]. Initially, we tried to conduct the reaction of 2-cyanobenzaldehyde (**2**) with CF_3_–SiMe_3_ in the presence of cinchona alkaloids **9**/TMAF combination (Table 2, entries 3–5). By employing catalyst **9b**, the reaction proceeded at −60 °C to give phthalide **1a** in 51% yield with 12% ee (Table 2, entry 4).

**Scheme 5:**
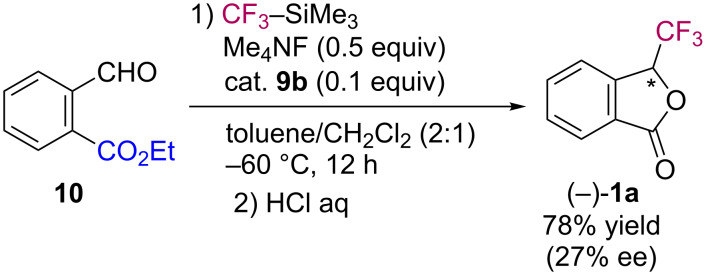
Catalytic asymmetric synthesis of **1a**.

To improve the enantioselectivity of the present nucleophilic trifluorometylation/lactonization, we surveyed suitable conditions for the catalytic asymmetric transformation. After many experiments, we found that the use of ethyl 2-formylbenzoate (**10**) [36] instead of nitrile **2** resulted in the formation of **1a** with 27% ee upon exposure to organocatalyst **9b** (0.1 equiv) and tetramethylammonium fluoride (0.5 equiv, Scheme 5).

## Conclusion

In summary, we have demonstrated a convenient route to fluorinated phthalides from 2-cyanobenzaldehyde or 2-formylbenzoates in a one-pot manner. In the transformations, installation of fluorinated substituents at the C3-position of phthalides has been achieved. The issue of the low stereoselectivity of the catalytic asymmetric fluoroalkylation should be solved in the future. Further examples for the promising utilization of fluorinated phthalides as building blocks can be found in [37].

## Supporting Information

File 1 General methods, synthetic procedures, ^1^H and ^19^F NMR spectra for known compound **1a** and full characterization of all new compounds.
