# Supplementary material for: Nucleophilic fluoroalkylation/cyclization route to fluorinated phthalides
Source: Beilstein J Org Chem. 2018 Jan 19;14:182–6. doi: 10.3762/bjoc.14.12 (PMC5789380; doi:10.3762/bjoc.14.12)
Supplement: File 1 — General methods, synthetic procedures, 1H and 19F NMR spectra for known compound 1a and full characterization of all new compounds. [file Beilstein_J_Org_Chem-14-182-s001.pdf]

Supporting Information

for

Nucleophilic fluoroalkylation/cyclization route to fluorinated phthalides

Masanori Inaba[1], Tatsuya Sakai[1], Shun Shinada[1], Tsuyuka Sugiishi[1], Yuta Nishina[2], Norio Shibata[3], and Hideki Amii*[1]

Address: [1]Division of Molecular Science, Graduate School of Science and Technology, Gunma University, 1-5-1 Tenjin-cho, Kiryu, Gunma, 376-8515, Japan, [2]Research Core for Interdisciplinary Sciences, Okayama University, 3-1-1 Tsushimanaka, Kita-ku, Okayama 700-8530, Japan and [3]Department of Nanopharmaceutical Sciences, Department of Life Science and Applied Chemistry, Nagoya Institute of Technology Gokiso, Showa-ku, Nagoya 466-8555, Japan

Email: Hideki Amii - amii@gunma-u.ac.jp

* Corresponding author

General methods, synthetic procedures, ^{1}H and ^{19}F NMR spectra for known compound 1a and full characterization of all new compounds

Contents

1. General remarks S2

2. Experimental procedures S2

3. References S5

4. Spectral characterization data S6

1. General remarks

All reactions were carried out under a nitrogen atmosphere in flame-dried glassware. Syringes which were used to transfer anhydrous solvents or reagents were purged with argon prior to use. Starting materials, reagents, and dry solvent were purchased from commercial suppliers and used without further purification. ^{1}H NMR spectra were recorded using Me_4Si as an internal standard (δ 0 ppm). ^{19}F NMR spectra were recorded in $CDCl_3$ on JEOL JNM-ECS300 (300 MHz), JEOL JNM-ECS400 (400 MHz) and JEOL JNM-ECA600 (600 MHz) spectrometers using $CFCl_3$ as an internal standard (δ 0 ppm). Splitting patterns were reported as s, singlet; d, doublet; t, triplet; q, quartet; m, multiplet; br, broad.

2. Experimental procedures

3-(Trifluoromethyl)phthalide (1a) [CAS Registry No. 76284-62-3] [1]

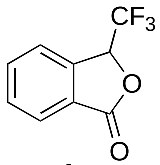

1a

Conditions A: To a mixture of 2-cyanobenzaldehyde (65.6 mg, 0.50 mmol), KF (20.9 mg, 0.36 mmol), and DMF (2.5 mL) was added trifluoromethyl(trimethyl)silane (85.3 mg, 0.60 mmol) at room temperature. The reaction mixture was stirred at room temperature in an atmosphere of nitrogen for 1 h and quenched with 1 M HCl (2.5 mL). The reaction mixture was stirred at 100 °C for 6 h. The aqueous layer was extracted with ethyl acetate. Then, the combined organic phase was washed with water, dried over anhydrous Na_2SO_4. Purification

by column chromatography on silica gel (hexane/ethyl acetate = 5/1) gave 95.9 mg (0.474 mmol, 95%) of 3-(trifluoromethyl)phthalide (**1a**) as a colorless oil; Mp 40.2-41.6 °C; IR (neat) 1780 cm^{-1}; ^{1}H NMR (400 MHz, $CDCl_3$) δ 5.68 (1H, q, *J* = 5.7 Hz), 7.66-7.72 (2H, m), 7.78-7.82 (1H, m), 8.00 (1H, d, *J* = 7.6 Hz); ^{19}F NMR (376 MHz, $CDCl_3$) δ -77.0 (3F, s); EI-MS *m/z* (%) 202 (M^+, 1), 155 (6), 133 (100), 105 (19), 77 (14).

3-(Pentafluoroethyl)phthalide (1b)

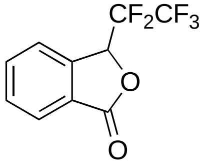

1b

The title compound was prepared according to the procedure A using 2-cyanobenzaldehyde (65.6 mg, 0.50 mmol), KF (20.9 mg, 0.36 mmol), and DMF (2.5 mL) was added pentafluoroethyl(trimethyl)silane (115.3 mg, 0.60 mmol) and purified by column chromatography (hexane/ethyl acetate = 5/1) to give colorless solid (97.0 mg, 0.385 mmol, 77%); Mp 60.8-62.9 °C; IR (neat) 1775 cm^{-1}; ^{1}H NMR (400 MHz, $CDCl_3$) δ 5.76 (1H, dd, *J* = 2.4, 17.6 Hz), 7.65-7.73 (2H, m), 7.79-7.83 (1H, m), 8.00 (1H, d, *J* = 7.6 Hz); ^{13}C NMR (150 MHz, $CDCl_3$) δ 74.9 (dd, *J* = 25.8, 33.0 Hz), 111.5 (ddq, *J* = 261, 253, 37.8 Hz), 118.3 (qt, *J* = 286, 34.5 Hz), 124.0 (d, *J* = 2.9 Hz), 126.0, 126.4, 131.2, 134.9, 140.5, 168.1; ^{19}F NMR (376 MHz, $CDCl_3$) δ -130.6 (1F, dd, *J* = 17.6, 282 Hz), -122.4 (1F, d, *J* = 282 Hz), -82.8--81.9 (3F, m); EI-MS *m/z* (%) 252 (M^+, 1), 205 (8), 133 (100), 105 (91), 77 (96). Anal. Calcd for $C_{10}H_5O_2F_5$: C, 47.64; H, 2.00. Found: C, 47.50; H, 2.17.

3-(Pentafluorophenyl)phthalide (1d)

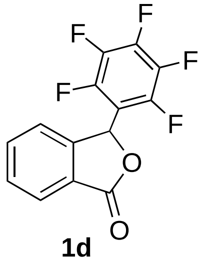

1d

The title compound was prepared according to the procedure A using 2-cyanobenzaldehyde (65.6 mg, 0.50 mmol), KF (20.9 mg, 0.36 mmol), and DMF (2.5 mL) was added pentafluorophenyl(trimethyl)silane (144.2 mg, 0.60 mmol) and purified by column chromatography (hexane/ethyl acetate = 5/1) to give colorless solid (129.6 mg, 0.432 mmol, 86%); Mp 110.0-112.6 °C; IR (neat) 1760 cm^{-1}; ^{1}H NMR (400 MHz, $CDCl_3$) δ 6.74 (1H, s), 7.34 (1H, d, *J* = 8.4 Hz), 7.61 (1H, t, *J* = 7.6 Hz), 7.70-7.74 (1H, m), 8.00 (1H, d, *J* = 7.6 Hz); ^{13}C NMR (75 MHz, $CDCl_3$) δ 71.9, 110.3 (t, *J* = 12.0 Hz), 122.0, 125.6, 126.1, 130.1, 134.7, 137.8 (d, *J* = 245 Hz), 145.0 (d, *J* = 250 Hz), 145.5 (d, *J* = 250 Hz),

146.7, 169.3; ^{19}F NMR (376 MHz, $CDCl_3$) δ -160.6--160.5 (2F, m), -151.0 (1F, t, *J* = 23.0 Hz), -142.8 (2F, dd, *J* = 6.0, 23.0 Hz); EI-MS *m/z* (%) 300 (M^+, 1), 255 (9), 237 (19), 133 (11), 105 (100), 77 (23). Anal. Calcd for $C_{14}H_5O_2F_5$: C, 56.02; H, 1.68. Found: C, 55.95; H, 1.77.

3-(Heptafluoropropyl)phthalide (1c)

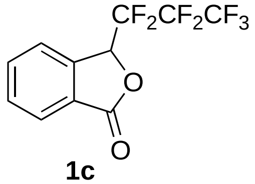

1c

Conditions B: To a mixture of 2-cyanobenzaldehyde (65.6 mg, 0.50 mmol), triethylamine (50.5 mg, 0.50 mmol), and DMF (2.5 mL) was added heptafluoropropyl(trimethyl)silane (145.3 mg, 0.60 mmol) at room temperature. The reaction mixture was stirred at room temperature in an atmosphere of nitrogen for 1 h and quenched with 4 M HCl (2.5 mL). The reaction mixture was stirred at 100 °C for 1 h. The aqueous layer was extracted with ethyl acetate. Then, the combined organic phase was washed with water, dried over anhydrous Na_2SO_4. Purification by column chromatography on silica gel (hexane/ethyl acetate = 5/1) gave 90.5 mg (0.300 mmol, 60%) of 3-(heptafluoropropyl)phthalide (**1c**) as colorless solid; Mp 45.0-46.2 °C; IR (neat) 1782 cm^{-1}; ^{1}H NMR (400 MHz, $CDCl_3$) δ 5.83 (1H, d, *J* = 18.4 Hz), 7.68-7.73 (2H, m), 7.78-7.82 (1H, m), 8.00 (1H, d, *J* = 7.6 Hz); ^{13}C NMR (150 MHz, $CDCl_3$) δ 75.0 (dd, *J* = 26.0, 34.0 Hz), 108.8 (tq, *J* = 265, 36.7 Hz), 113.3 (ddt, *J* = 252, 264, 30.7 Hz), 117.5 (qt, *J* = 286, 33.0 Hz), 124.2, 126.1, 126.3, 131.2, 134.9, 140.5, 168.2; ^{19}F NMR (376 MHz, $CDCl_3$) δ -127.2 (1F, d, *J* = 298 Hz), -126.6 (1F, d, *J* = 291 Hz), -125.7 (1F, d, *J* = 291 Hz), -120.2 (1F, d, *J* = 298 Hz), -80.9 (3F, t, *J* = 15.4 Hz); EI-MS *m/z* (%) 302 (M^+, 1), 169 (7), 133 (100), 105 (63), 77 (78). Anal. Calcd for $C_{14}H_5O_2F_5$: C, C 43.73; H, 1.67. Found: C, 43.29; H, 1.81.

(−)-3-(Trifluoromethyl)phthalide ((−)-1a)

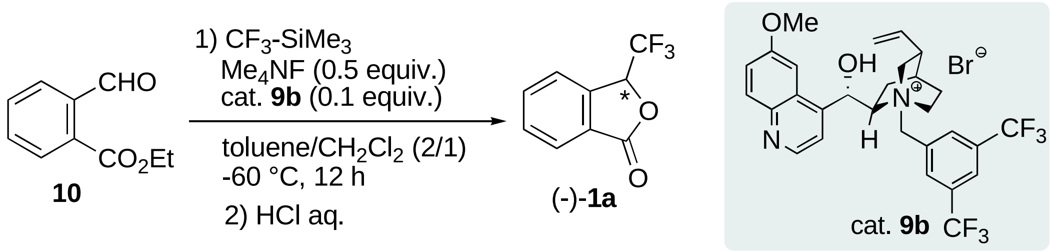

To a mixture of ethyl 2-formylbenzoate (**10**; 89.1 mg, 0.50 mmol), **9b** (15.8 mg, 0.025 mmol),[2] tetramethylammonium fluoride (9.3 mg, 0.10 mmol), toluene (4.0 mL), and dichloromethane (2.0 mL) was added trifluoromethyl(trimethyl)silane (141.3 mg, 1.0 mmol) at −60 °C. The reaction mixture was stirred at room temperature in an atmosphere of

nitrogen at −60 °C for 24 h and quenched with sat. NH_4Cl aq (5 mL). The aqueous layer was extracted with ethyl acetate. Then, the combined organic phase was washed with water, dried over anhydrous Na_2SO_4. After removal of the solvent under reduced pressure, to the residue was added DMF (2.5 mL) and 1 M HCl (2.5 mL). The reaction mixture was stirred at 100 °C for 6 h. The aqueous layer was extracted with ethyl acetate. Then, the combined organic phase was washed with water, dried over anhydrous Na_2SO_4. Purification by column chromatography on silica gel (hexane/ethyl acetate = 5:1) gave 78.8 mg (0.39 mmol, 78% yield, 27% ee) of 3-(trifluoromethyl)phthalide (**1a**) as a colorless oil; ^{1}H NMR (400 MHz, $CDCl_3$) δ 5.68 (1H, q, J = 5.7 Hz), 7.66-7.72 (2H, m), 7.78-7.82 (1H, m), 8.00 (1H, d, J = 7.6 Hz); HPLC condition: Chiralcel OD-H, hexane/2-propanol = 9/1, flow = 1.0 mL/min, wavelength = 224 nm, temp. = 30 °C, t_R = 8.5 min for (-)-**1a** and t_R = 12.0 min for (+)-**1a**. [1]

3. References

1) R. Pedrosa, S. Sayalero, M. Vicente, *Tetrahedron* **2006**, *62*, 10400.
2) S. Mizuta, N. Shibata, S. Akiti, H. Fujimoto, S. Nakamura, T. Toru, *Org. Lett.* **2007**, *9*, 3707.

4. Spectral Characterization Data

^{1}H NMR Spectrum of 3-(trifluoromethyl)phthalide (**1a**)

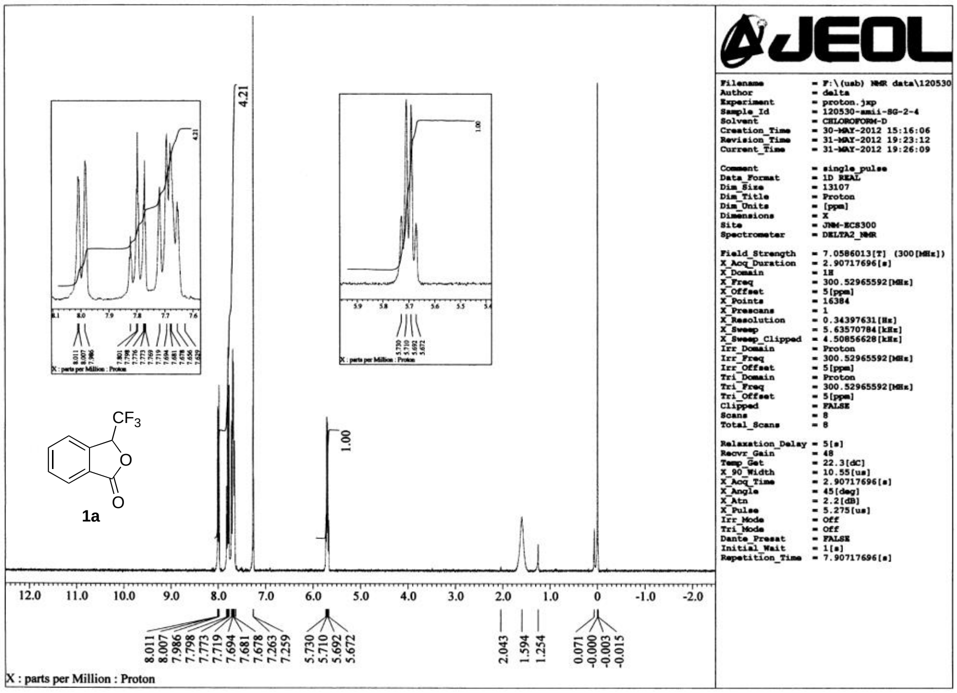

^{19}F NMR Spectrum of 3-(trifluoromethyl)phthalide (**1a**)

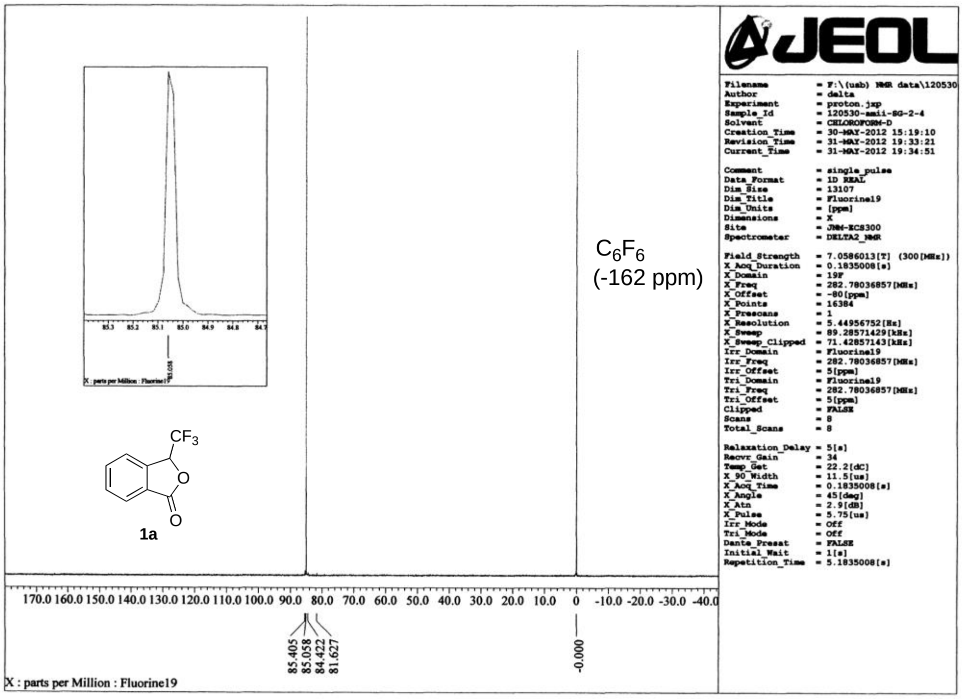

^{1}H NMR Spectrum of 3-(pentafluoroethyl)phthalide (**1b**)

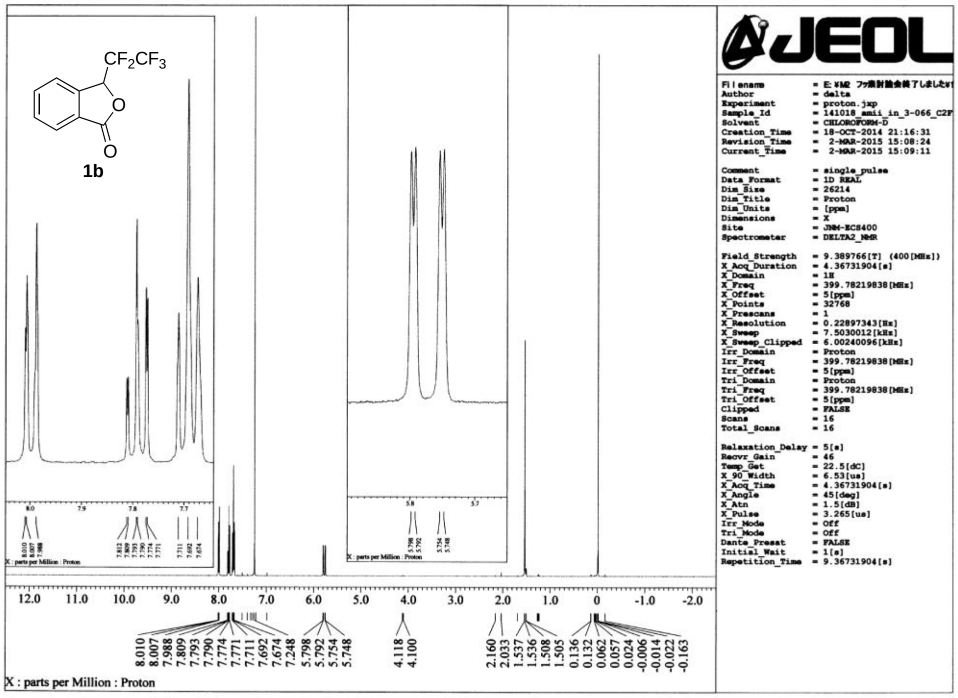

^{13}C NMR Spectrum of 3-(pentafluoroethyl)phthalide (**1b**)

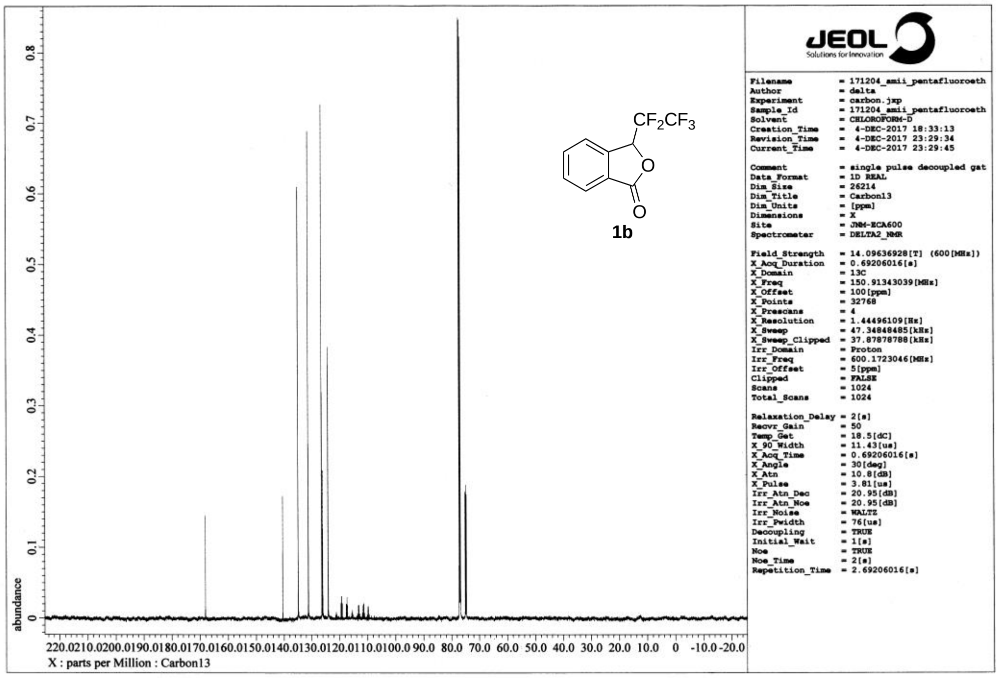

^{19}F NMR Spectrum of 3-(pentafluoroethyl)phthalide (**1b**)

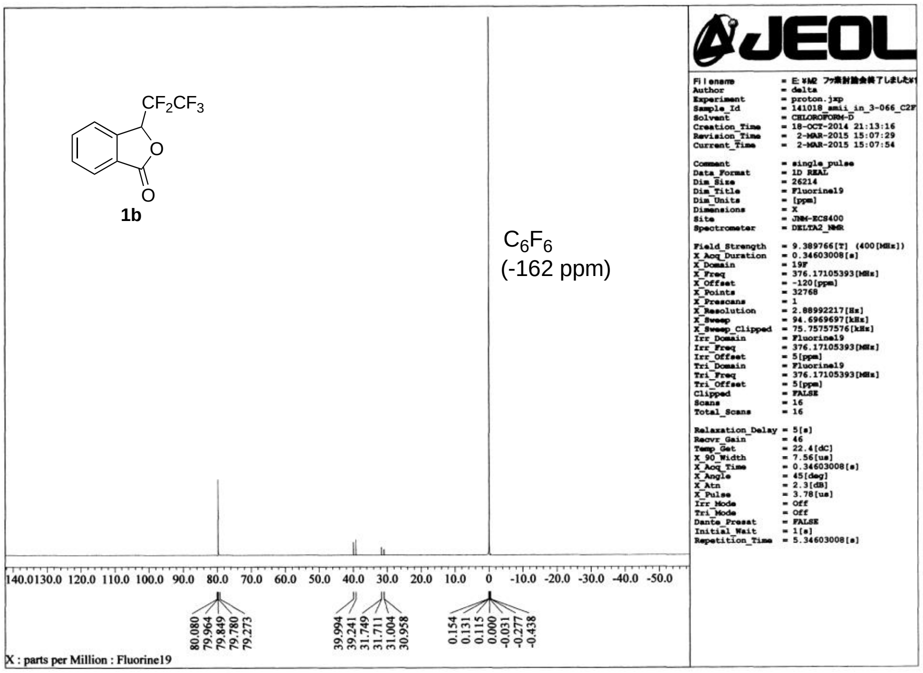

^{1}H NMR Spectrum of 3-(heptafluoropropyl)phthalide (**1c**)

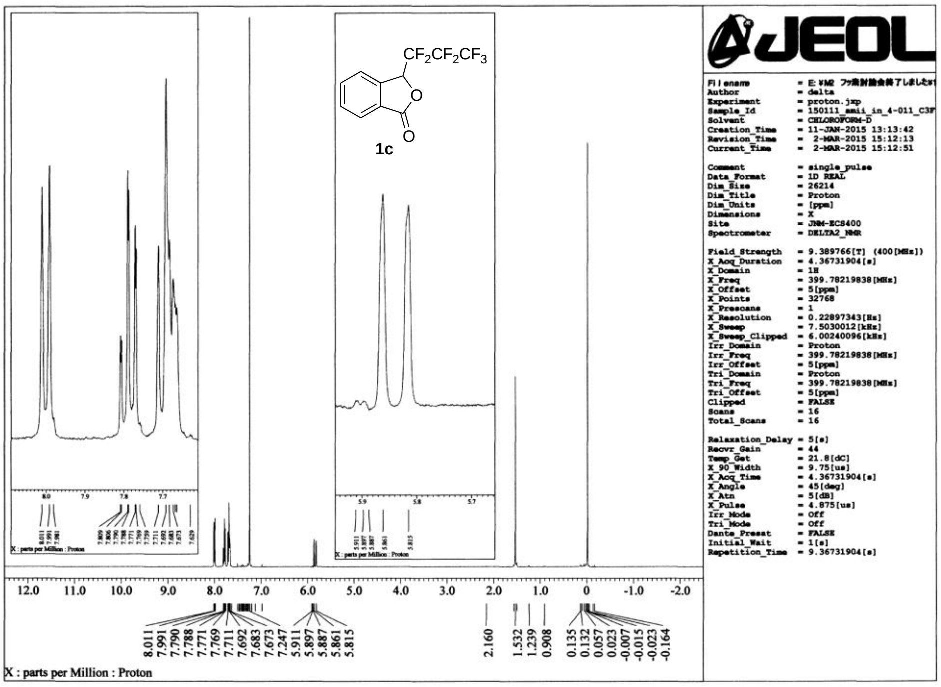

^{13}C NMR Spectrum of 3-(heptafluoropropyl)phthalide (**1c**)

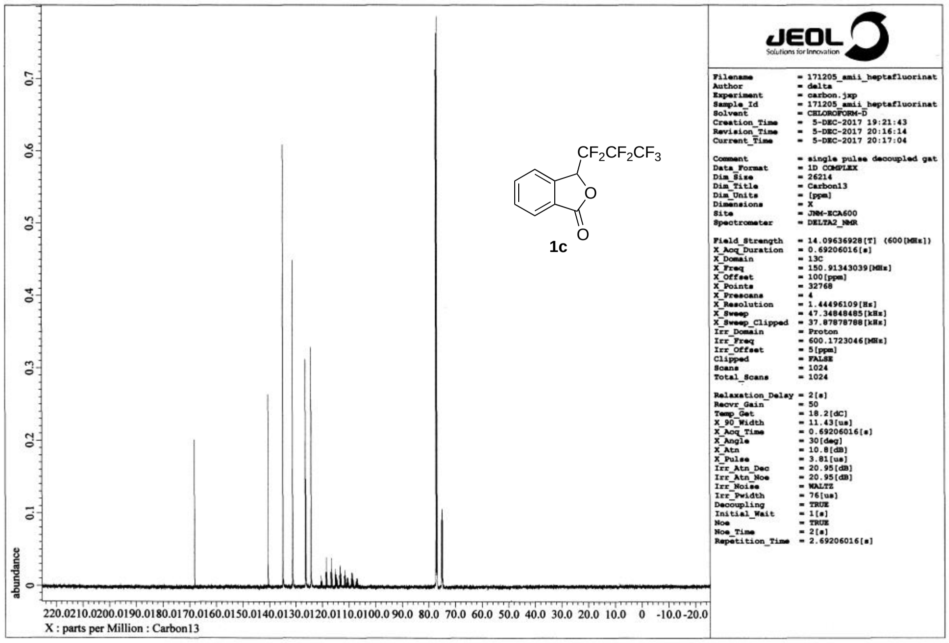

^{19}F NMR Spectrum of 3-(heptafluoropropyl)phthalide (**1c**)

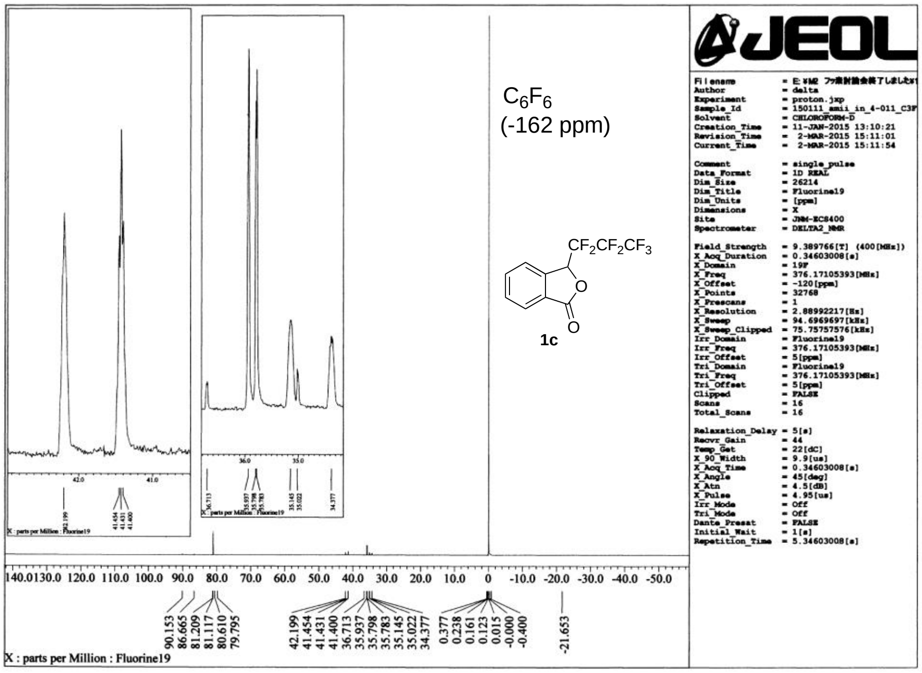

^{1}H NMR Spectrum of 3-(pentafluorophenyl)phthalide (**1d**)

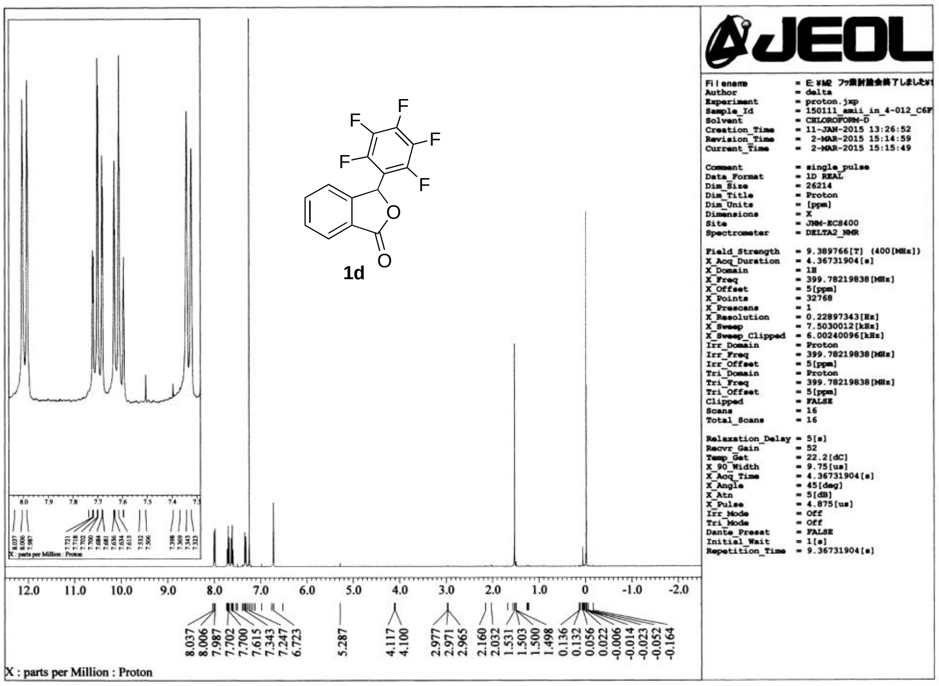

^{13}C NMR Spectrum of 3-(pentafluorophenyl)phthalide (**1d**)

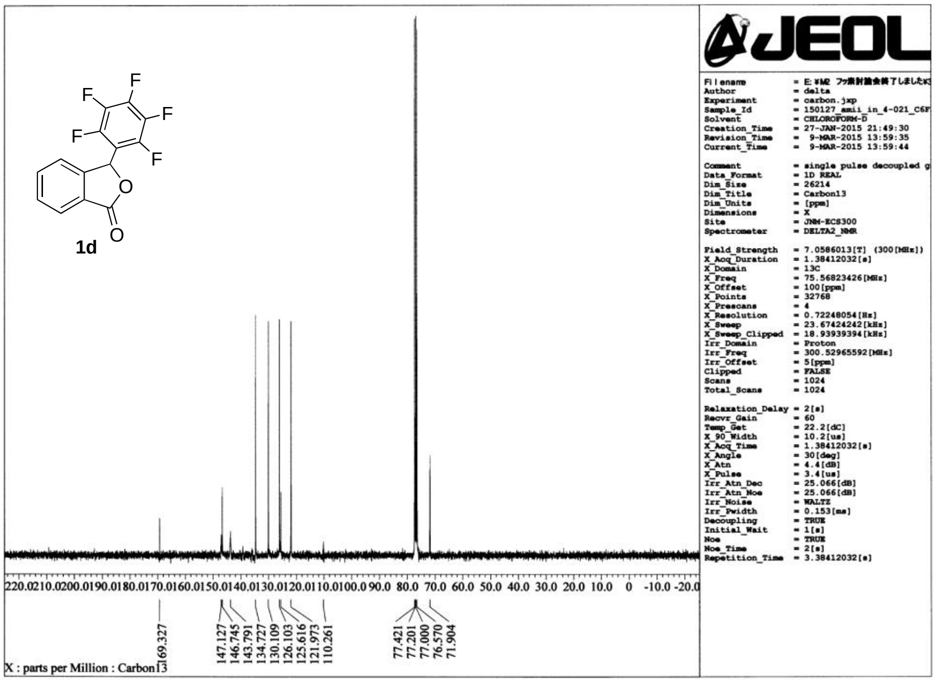

^{19}F NMR Spectrum of 3-(pentafluorophenyl)phthalide (**1d**)

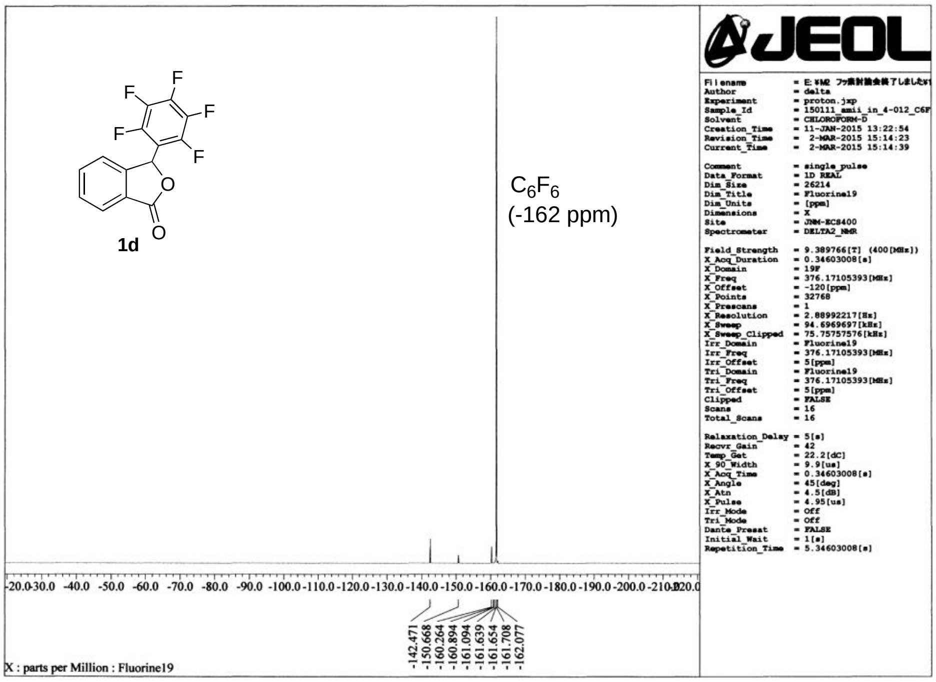

HPLC Analytical Data of 3-(trifluoromethyl)phthalide (**1a**)

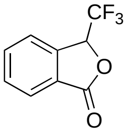

rac-**1a**

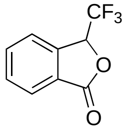

(-)-**1a**
(27% ee)

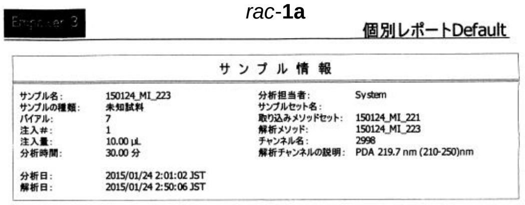

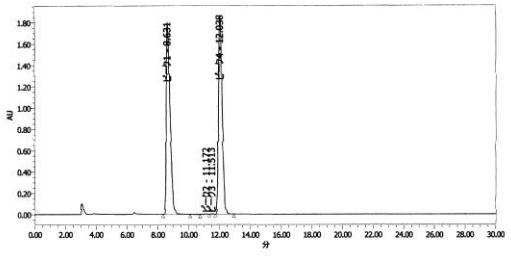

	成分名	保持時間 (分)	面積 (µV秒)	%面積	高さ (µV)
1	ピーク1	8.631	31013021	49.98	1798444
2	ピーク2	11.172	51819	0.08	3535
3	ピーク3	11.513	42631	0.07	2594
4	ピーク4	12.038	30942364	49.87	1863154

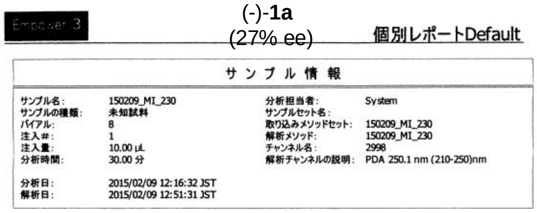

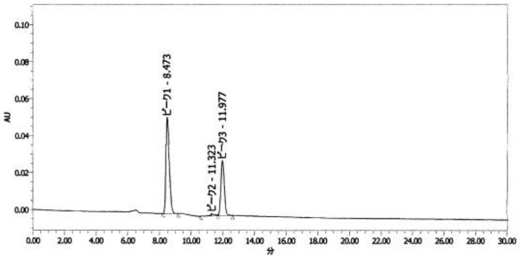

	成分名	保持時間 (分)	面積 (µV秒)	%面積	高さ (µV)
1	ピーク1	8.473	785284	62.18	52147
2	ピーク2	11.323	25760	2.04	1554
3	ピーク3	11.977	451785	35.78	29986
